# Genetic and Epigenetic Variations Induced by Wheat-Rye 2R and 5R Monosomic Addition Lines

**DOI:** 10.1371/journal.pone.0054057

**Published:** 2013-01-14

**Authors:** Shulan Fu, Chuanfei Sun, Manyu Yang, Yunyan Fei, Feiqun Tan, Benju Yan, Zhenglong Ren, Zongxiang Tang

**Affiliations:** State Key Laboratory of Plant Breeding and Genetics, Sichuan Agricultural University, Wenjiang, Chengdu, Sichuan, People’s Republic of China; Nanjing Agricultural University, China

## Abstract

**Background:**

Monosomic alien addition lines (MAALs) can easily induce structural variation of chromosomes and have been used in crop breeding; however, it is unclear whether MAALs will induce drastic genetic and epigenetic alterations.

**Methodology/Principal Findings:**

In the present study, wheat-rye 2R and 5R MAALs together with their selfed progeny and parental common wheat were investigated through amplified fragment length polymorphism (AFLP) and methylation-sensitive amplification polymorphism (MSAP) analyses. The MAALs in different generations displayed different genetic variations. Some progeny that only contained 42 wheat chromosomes showed great genetic/epigenetic alterations. Cryptic rye chromatin has introgressed into the wheat genome. However, one of the progeny that contained cryptic rye chromatin did not display outstanding genetic/epigenetic variation. 78 and 49 sequences were cloned from changed AFLP and MSAP bands, respectively. Blastn search indicated that almost half of them showed no significant similarity to known sequences. Retrotransposons were mainly involved in genetic and epigenetic variations. Genetic variations basically affected *Gypsy*-like retrotransposons, whereas epigenetic alterations affected *Copia*-like and *Gypsy*-like retrotransposons equally. Genetic and epigenetic variations seldom affected low-copy coding DNA sequences.

**Conclusions/Significance:**

The results in the present study provided direct evidence to illustrate that monosomic wheat-rye addition lines could induce different and drastic genetic/epigenetic variations and these variations might not be caused by introgression of rye chromatins into wheat. Therefore, MAALs may be directly used as an effective means to broaden the genetic diversity of common wheat.

## Introduction

Modern cultivation procedures have narrowed the genetic base of common wheat (*Triticum aestivum* L.). A lot of wild relatives and related species were widely used to increase the genetic diversity available to wheat breeders. Rye (*Secale cereale* L.) has great potential for expanding the genetic variability of cultivated common wheat. Some newly synthesized triticale have been particularly revealing of rapid genomic and epigenomic changes [1]–[4]. The structural variations of rye chromosomes in triticale were also discovered [5]–[8]. Triticales are the beginning of introgression of rye chromatin into wheat genome and they were used to produce wheat-rye addition, substitution and translocation lines. Wheat-rye addition, substitution and translocation lines are the direct bridge materials for wheat improvement. The advantages of using addition lines are the possibility of locating alien gene(s) or species-specific characteristics to particular chromosomes, and the potential to transfer elite gene(s) between species. The alterations of rye telomeric/subtelomeric heterochromatin were observed in several sets of wheat-rye disomic addition lines [9]. Chromosome instability and genome rearrangements in wheat-rye disomic addition lines were also observed [10]–[11]. Almost all these previous studies focused on the wheat-rye disomic addition lines. Researchers should pay more attentions to monosomic alien addition lines (MAALs). It is an effective method to use MAALs to induce small-segment-translocation between species [12]. In rice (*Oryza sativa* L) breeding program, some useful genes were transferred into cultivated rice from its related wild species by MAALs [13]–[14]. MAALs were also used in breeding programs of sugar beet, oilseed rape, wheat and onion [12], [15]–[17]. Although many MAALs were used in crop breeding, it is unclear whether single alien chromosome added to plants can induce drastic genetic and epigenetic alterations. In present study, wheat-rye 2R and 5R monosomic addition lines and their selfed progeny were used to investigate the effects of single rye chromosome on the alterations of wheat genome.

## Results

### Identification of Wheat-rye Monosomic Addition Lines

**Figure 1:**
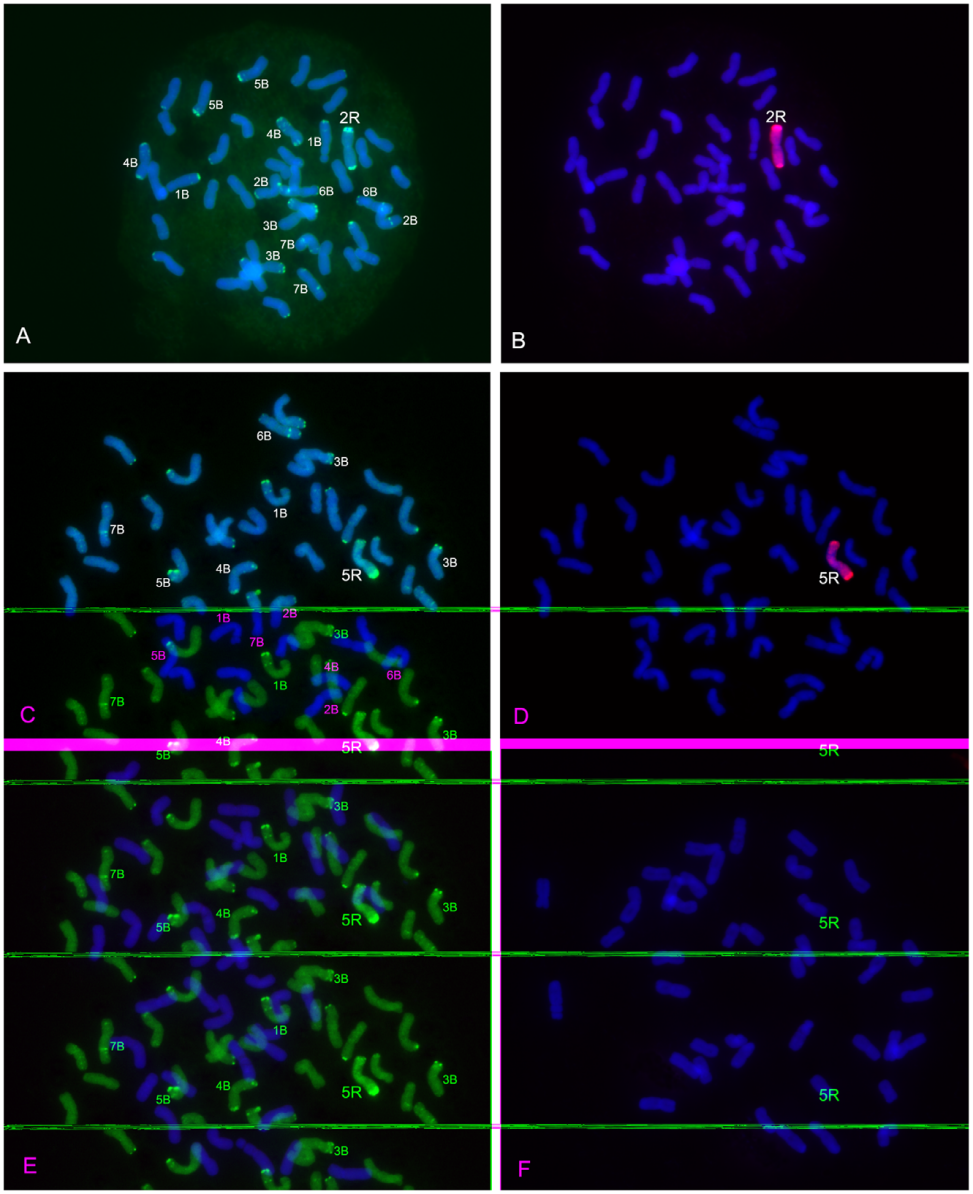
Wheat-rye monosomic addition lines and their progeny were identified by FISH and GISH analyses using pSc119.2 and rye genomic DNA as probes. **A, B** Sequential FISH and GISH on the same spread of metaphase chromosomes of root tip of MY+2RF_3_ representing 2R monosomic addition line. 2R chromosome and B-genomic chromosomes were marked. **C, D** Sequential FISH and GISH on the same spread of metaphase chromosomes of root tip of MY+5RF_3_ representing 5R monosomic addition line. 5R chromosome and B-genomic chromosomes were marked. **E** GISH analysis on 2RF_3_MY-4. **F** GISH analysis on 5RF_3_MY-1. **E** and **F** represent the results of GISH analysis on the progeny that only contained 42 wheat chromosomes.

**Figure 2:**
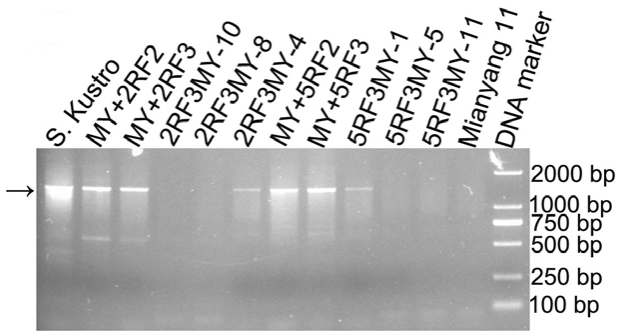
PCR amplification using rye-specific marker. Rye-specific marker was used to investigate whether small rye chromatin has introgressed into the progeny that only contained 42 wheat chromosomes. Arrow indicates the target bands.

From BC_1_F_2_ seeds, one wheat-rye 2R monosomic addition line and one wheat-rye 5R monosomic addition line were identified (Figure 1). The 2R and 5R monosomic addition lines were respectively named as MY+2RF_2_ and MY+5RF_2_. Then the 2R and 5R monosomic addition lines were bagged for self-fertilization. Twenty-two seeds (BC_1_F_3_) from the selfed progeny of MY+2RF_2_ and 17 seeds (BC_1_F_3_) from the selfed progeny of MY+5RF_2_ germinated. A 2R monosomic addition line (MY+2RF_3_) and a 5R monosomic addition line (MY+5RF_3_) were respectively identified from the selfed progeny of MY+2RF_2_ and MY+5RF_2_ (Figure 1). GISH analysis did not detect the translocations between wheat and rye chromosomes in MY+2RF_2_, MY+2RF_3_, MY+5RF_2_ and MY+5RF_3_ (Figure 1). The other 37 plants derived from MY+2RF_2_ and MY+5RF_2_ only contained 42 wheat chromosomes and they were named as 2RF_3_MY and 5RF_3_MY, respectively. ‘Mianyang11’, MY+2RF_2_, MY+2RF_3_, MY+5RF_2_ and MY+5RF_3_ were used for AFLP and MSAP analyses. In addition, 2RF_3_MY-4, 2RF_3_MY-8, 2RF_3_MY-10, 5RF_3_MY-1, 5RF_3_MY-5 and 5RF_3_MY-11 were randomly selected from the 37 plants. Although the parental rye ‘Kustro’ was kept by selfing, it was not used for AFLP and MSAP analyses because of its possible heterogeneity. GISH analysis did not detect small translocations between wheat and rye chromosomes in 2RF_3_MY-4, 2RF_3_MY-8, 2RF_3_MY-10, 5RF_3_MY-1, 5RF_3_MY-5 and 5RF_3_MY-11 (Figure 1). The result indicated that the six progeny only contained 42 wheat chromosomes. However, PCR reaction using rye-specific marker produced the target bands in 2RF_3_MY-4 and 5RF_3_MY-1 (Figure 2). These results indicated that rye chromatins have already introgressed into wheat genome and they can not be detected by GISH analysis. 2RF_3_MY-4 and 5RF_3_MY-1 were still considered as the progeny that only contained 42 wheat chromosomes because they just contained cryptic rye chromatins.

### AFLP Analysis

**Figure 3:**
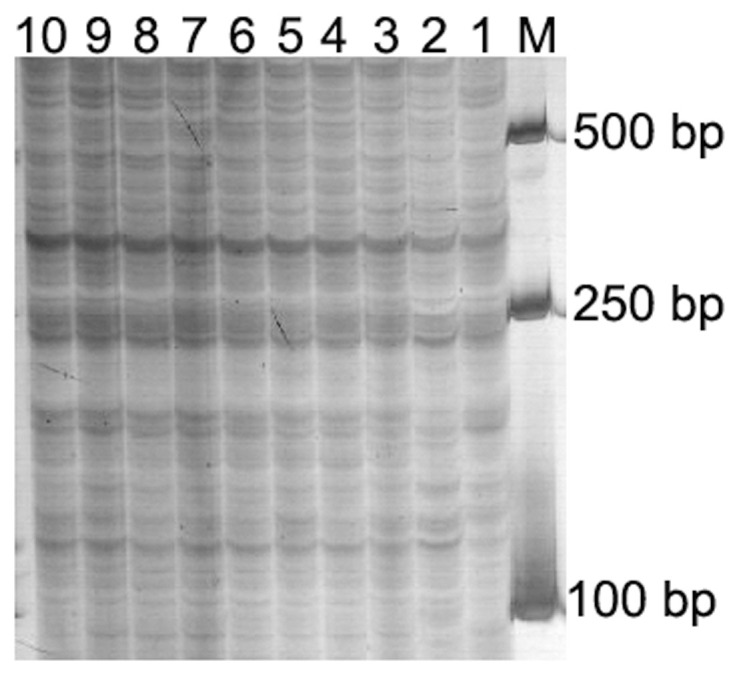
Method of AFLP was used to analyze the ten parental wheat plants. AFLP fingerprints of genomic DNA of ten ‘Mianyang 11’ plants displayed by selective primer combination *Eco*R I-ACG+ *Mse* I-CAA. M, DNA marker. 1–10, ten single ‘Mianyang 11’ plants.

**Figure 4:**
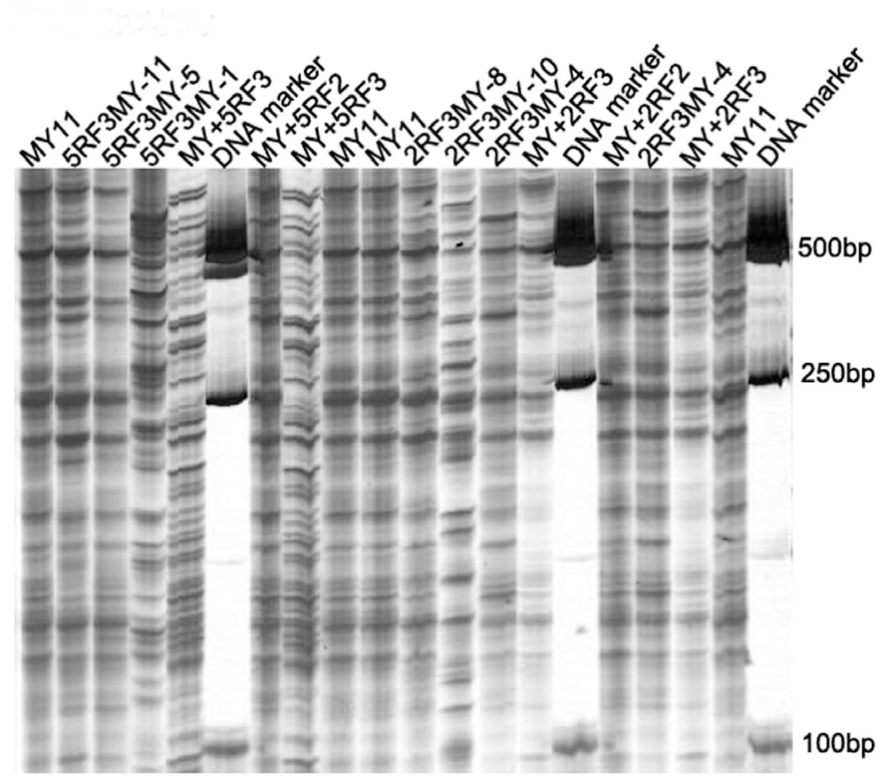
Method of AFLP was used to analyze the 11 materials in present study. AFLP fingerprints of genomic DNA of the 11 materials displayed by selective primer combination *Eco*R I-AAC+ *Mse* I-CTG. MY11, ‘Mianyang11’.

Eight different AFLP selective primer combinations (Table 1) were first used to investigate the genetic variations of ten ‘Mianyang11’ plants. Almost no polymorphic amplified sites were found among the ten ‘Mianyang11’ plants (Figure 3). This result indicated that parental wheat plants were homozygous. Using the eight AFLP selective primer combinations, a total of 3065 bands were obtained from ‘Mianyang 11’ and its ten progeny used in this study. Polymorphic amplified sites were observed among the 11 materials (Figure 4). The numbers of absent parental bands and new bands in some progeny were listed in Table 2. Compared with parental wheat ‘Mianyang 11’, a total of 1135 changed bands including 531 lost and 604 new bands were detected in its six progeny, which only contained 42 wheat chromosomes (Table 2). Compared with MY+2RF_2_ (2R monosomic addition line, BC_1_F_2_), a total of 809 changed bands including 500 lost and 309 new bands were detected in its four selfed progeny (Table 2). When MY+5RF_2_ (5R monosomic addition line, BC_1_F_2_) was compared with its four selfed progeny, a total of 805 changed bands including 447 lost and 358 new bands were observed (Table 2). Great genetic variations were observed among the six progeny that only contained 42 wheat chromosomes (F = 9.10; F_0.05_ = 2.89). 2RF_3_MY-10 and 5RF_3_MY-1 exhibited great genetic variations when they were respectively compared with ‘Mianyang 11’ (t_2RF3MY-10_ = 3.41; t_5RF3MY-1_ = 3.88; t_0.05_ = 2.365). There were great genetic variations between MY+2RF_2_ and MY+2RF_3_ (t = 2.72; t_0.05_ = 2.365), and between MY+5RF_2_ and MY+5RF_3_ (t = 3.42; t_0.05_ = 2.365). Different genetic variations could be also observed when MY+2RF_2_ was compared with its selfed progeny and MY+5RF_2_ was compared with its selfed progeny (Table 2). These results indicated that wheat-rye monosomic addition lines could induce different and drastic genetic variation in their progeny.

**Table 1:** Sequences of AFLP and MSAP adapters and primers used in this study.

Adaptors	
*Eco*R I-adaptors-F	5′CTCGTAGACTGCGTACC3′
*Eco*R I-adaptors-R	5′AATTGGTACGCAGTC3′
*Mse* I-adaptors-F	5′GACGATGAGTCCTGAG3′
*Mse* I-adaptors-R	5′TACTCAGGACTCAT3′
*Hpa* II/*Msp* I-adaptors-F	5′GATCATGAGTCCTGCT3′
*Hpa* II/*Msp* I-adaptors-R	5′CGAGCAGGACTCATGA3′
Preselective primers	
*Eco*R I-**A**	5′GACTGCGTACCAATTC**A**3′
*Mse* I-**C**	5′GACGATGAGTCCTGAGTAA**C**3′
*Hpa* II/*Msp* I-**T**	5′ATCATGAGTCCTGCTCGG**T**3′
**Selective primer combinations used in AFLP**	
*Eco*R I-**AAC**+ *Mse* I-**CTC**	*Eco*R I-**ACC**+ *Mse* I-**CAA**
*Eco*R I-**AAC**+ *Mse* I-**CTG**	*Eco*R I-**AGG**+ *Mse* I-**CAA**
*Eco*R I-**ACT**+ *Mse* I-**CTG**	*Eco*R I-**ACG**+ *Mse* I-**CAA**
*Eco*R I-**ACA**+ *Mse* I-**CAA**	*Eco*R I-**ACG**+ *Mse* I-**CAA**
**Selective primer combinations used in MSAP**	
*Eco*R I-**AAC**+ *Hpa* II/*Msp* I-**TTCT** (E1HM7)	*Eco*R I-**AAG**+ *Hpa* II/*Msp* I-**TTGC** (E2HM2)
*Eco*R I-**AAC**+ *Hpa* II/*Msp* I-**TCGA** (E1HM1)	*Eco*R I-**AAG**+ *Hpa* II/*Msp* I-**TCGA** (E2HM1)
*Eco*R I-**AAC**+ *Hpa* II/*Msp* I-**TCCA** (E1HM3)	

**Table 2:** Numbers of lost and new bands in progeny compared with their parental plants.

Materials	Number of lost and new bands compared with parents					
	Compared with ‘Mianyang 11’*		Compared with MY+2RF_2_ *		Compared with MY+5RF_2_ *	
	Number of lost bands	Number of new bands	Number of lost bands	Number of new bands	Number of lost bands	Number of new bands
‘Mianyang11’	–	–	–	–	–	–
MY+2RF_2_	–	–	–	–	–	–
MY+2RF_3_	–	–	136	62	–	–
2RF_3_MY-4	67	79	86	41	–	–
2RF_3_MY-8	93	75	118	75	–	–
2RF_3_MY-10	132	171	160	131	–	–
MY+5RF_2_	–	–	–	–	–	–
MY+5RF_3_	–	–	–	–	154	120
5RF_3_MY-1	132	113	–	–	150	109
5RF_3_MY-5	57	51	–	–	86	38
5RF_3_MY-11	50	115	–	–	57	91
Total	531	604	500	309	447	358

* ‘–’indicates no comparison was done.

### MSAP Analysis

**Figure 5:**
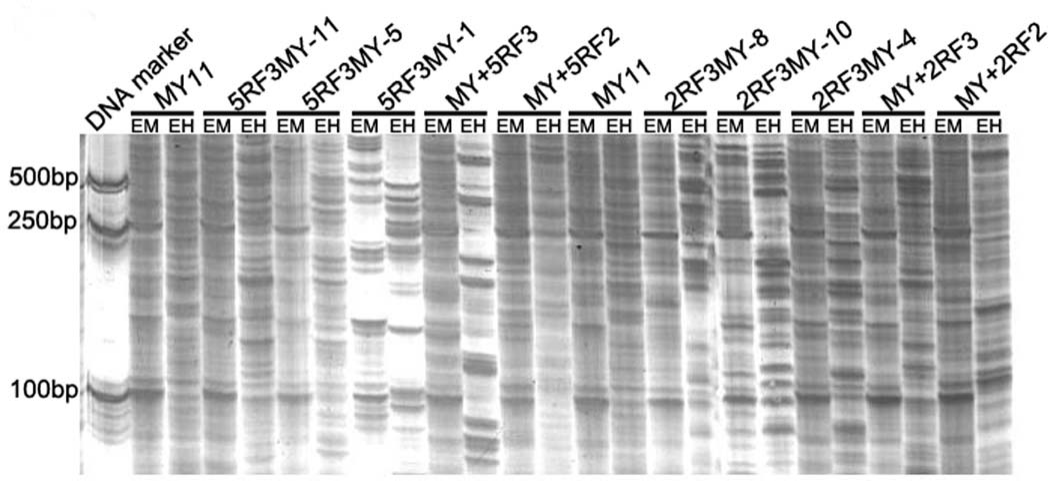
Method of MSAP was used to analyze the 11 materials in present study. MSAP fingerprints of genomic DNA of the 11 materials and variation of methylation pattern displayed by selective primer combination *Eco*R I-AAC+ *Hpa* II/*Msp* I-TCGA. EM, *Eco*R I+*Msp* I; EH, *Eco*R I+*Hpa* II; MY11, ‘Mianyang11’.

Because *Hpa*II will not cut if either the outer or the inner cytosine of the ‘CCGG/GGCC’ site is fully (double-strand) methylated, whereas, *Msp*I will not cut if the external cytosine is fully or hemi- (single-strand) methylated, the methylation states of the cytosine at ‘CCGG/GGCC’ sites would lead to a differential cleavage by the two isoschizomers. Thus, the band pattern from PCR amplification can reflect the methylation status at a certain site (Figure 5). The five pairs of *Eco*RI+*Hpa*II/*Msp*I selective primer combinations produced reproducible fragments at 134 sites (Table 3). For each of the materials used in this study, the genomic DNA methylation extent including full-methylation and hemi-methylation is list in Table 3. From Table 3, different methylation extent and pattern of the genomic DNA among the 11 materials can be observed. Among the six progeny that contained 42 wheat chromosomes, only 2RF_3_MY-10 and 5RF_3_MY-1 displayed great differences from ‘Mianyang 11’ in the extent of full methylation (t_2RF3MY-10_ = 3.10; t_5RF3MY-1_ = 3.27; t_0.05_ = 2.776), and only 5RF_3_MY-1 displayed great differences from ‘Mianyang 11’ in the extent of hemi-methylation (t = 2.90; t_0.05_ = 2.776). There was no great epigenetic variation between MY+2RF_2_ and MY+2RF_3_. The great epigenetic variation was not observed between MY+5RF_2_ and MY+5RF_3_, too. These results indicated that some of the progeny of monosomic addition lines displayed great epigenetic variations.

**Table 3:** Number of bands amplified using five MSAP selective primer combinations in the 11 materials used in this study.

Primer combinations*			E1HM7	E1HM1	E1HM3	E2HM2	E2HM1	Total		Rate of methylated site (%)	
Number of amplification site			31	19	26	27	31	134			
Mianyang11	Methylated site	Fully**	2	1	1	2	4	10	54	7.46	40.30
		Hemi***	11	4	12	9	8	44		32.84	
MY+2RF_2_	Methylated site	Fully	4	2	5	6	4	21	51	15.67	38.06
		Hemi	8	1	6	5	10	30		22.39	
MY+2RF_3_	Methylated site	Fully	2	3	7	5	5	22	56	16.42	41.79
		Hemi	7	2	6	6	13	34		25.37	
2RF_3_MY-4	Methylated site	Fully	3	4	5	8	5	25	61	18.66	45.52
		Hemi	10	6	3	4	13	36		26.87	
2RF_3_MY-8	Methylated site	Fully	1	1	3	8	8	21	65	15.67	48.51
		Hemi	13	4	12	7	8	44		32.84	
2RF_3_MY-10	Methylated site	Fully	7	3	7	5	7	27	63	20.15	47.01
		Hemi	8	6	8	7	7	36		26.87	
MY+5RF_2_	Methylated site	Fully	4	2	5	5	7	23	46	17.16	34.33
		Hemi	11	2	7	2	1	23		17.16	
MY+5RF_3_	Methylated site	Fully	8	4	5	9	8	34	63	25.37	47.01
		Hemi	10	1	4	4	10	29		21.64	
5RF_3_MY-1	Methylated site	Fully	8	1	3	10	8	30	52	22.39	38.81
		Hemi	6	2	4	5	5	22		16.42	
5RF_3_MY-5	Methylated site	Fully	4	1	3	4	2	14	52	10.45	38.81
		Hemi	6	0	11	6	15	38		28.36	
5RF_3_MY-11	Methylated site	Fully	4	0	4	3	8	19	65	14.18	48.51
		Hemi	6	12	9	8	11	46		34.33	

* The names of primer combinations are abbreviations and their corresponding full name are listed in Table 1.

** The band pattern that absent for *Hpa* II but present for *Msp* I was regarded as full-methylation and the numbers of this kind of band pattern amplified by each primer pairs were scored in each material.

*** The band pattern that for present *Hpa* II but absent for *Msp* I was regarded as hemi-methylation and the numbers of this kind of band pattern amplified by each primer pairs were scored in each material.

Among the methylated sites, no monomorphic sites were observed, that is, all the 134 methylated sites showed polymorphism among the 11 materials. According to the extent of methylation variation in ‘CCGG/GGCC’ site, the methylated site which showed polymorphism patterns could be further divided into three types: (i) hypermethylation polymorphism site (HPS), the extent of cytosine methylation in this site was stronger than parental plants; (ii) demethylation polymorphism site (DPS), the extent of cytosine methylation in this site was weaker than parental plants; (iii) uncertain polymorphism site (UPS), the extent of cytosine methylation in this site could not be accurately qualitative compared with parental plants. The types of polymorphic methylated sites were determined by using ‘Mianyang11’ as reference. The criteria that were used to judge the patterns of polymorphic methylated sites were listed in Table 4. The polymorphism patterns of methylated sites in the progeny were listed in Table 5. From the Table 5, it could be noted that the numbers of HPS and DPS sites were different among the ten progeny. The six progeny that only contained 42 wheat chromosomes displayed great differences in both HPS and DPS sites (F_HPS_ = 9.34; F_DPS_ = 4.58; F_0.05_ = 3.15), however, no significant differences were observed between MY+2RF_2_ and MY+2RF_3_ in HPS and DPS sites. Similarly, there were no great differences between MY+5RF_2_ and MY+5RF_3_ in HPS and DPS sites. All these results mentioned above indicated that wheat-rye monosomic addition lines could induce different and drastic epigenetic variation in their progeny that only contained 42 wheat chromosomes.

**Table 4:** Criteria by which patterns of polymorphic methylated sites were judged.

Methylation patterns		hypermethylationpolymorphism site (HPS)					demethylationpolymorphism site (DPS)					uncertainpolymorphism site (UPS)	
Parental plants*	EM	−	−	−	+	+	+	+	+	−	−	−	+
	EH	+	−	−	+	+	−	−	−	+	+	−	+
Progeny*	EM	+	−	+	−	+	−	−	+	−	+	+	−
	EH	−	+	−	+	−	+	−	+	−	+	+	−

* EM, *Eco*R I+*Msp* I; EH, *Eco*R I+*Hpa* II; -, band absent; +, band present.

**Table 5:** Types of polymorphic methylated sites in the ten progeny of ‘Mianyang11’.

Progeny	Polymorphicpattern*	Number of polymorphic site**
MY+2RF_2_	HPS	35
	DPS	34
	UPS	18
MY+2RF_3_	HPS	35
	DPS	30
	UPS	11
2RF_3_MY-4	HPS	45
	DPS	35
	UPS	13
2RF_3_MY-8	HPS	32
	DPS	22
	UPS	5
2RF_3_MY-10	HPS	44
	DPS	26
	UPS	15
MY+5RF_2_	HPS	31
	DPS	37
	UPS	15
MY+5RF_3_	HPS	46
	DPS	32
	UPS	8
5RF_3_MY-1	HPS	37
	DPS	38
	UPS	13
5RF_3_MY-5	HPS	31
	DPS	36
	UPS	6
5RF_3_MY-11	HPS	34
	DPS	23
	UPS	9

* HPS, hypermethylation polymorphism site; DPS, demethylation polymorphism site; UPS, unpone.0054057.g001.tifcertain polymorphism site.

** The numbers were obtained by comparing ‘Mianyang11’ with each of its ten progeny.

### Sequencing of Polymorphic AFLP and MSAP Fragments

Seventy-eight lost and new AFLP fragments were gel isolated, reamplified and sequenced (JX518989-JX519066). Blastn search in NCBI revealed the characterization of these sequences. For 39 of the 78 AFLP sequences, no significant similarities were found. Among the rest 39 sequences, one, seven, six and 19 sequences were respectively involved in tandem repeat, DNA transposon, *Copia*-like retrotransposon and *Gypsy*-like retrotransposon. In addition, three sequences had over 90% similarity to cDNA, one sequence, which was cloned from one of new bands in 5RF_3_MY-1, only matched (81% similarity) to *Secale cereale* clone R4-6 genomic sequence (DQ414511.1) and two sequences had over 90% similarity to known wheat genomic DNA sequences that were anonymous.

Forty-nine MSAP fragments that displayed methylation alteration were also gel isolated, reamplified and sequenced (JX519067-JX519115). Blastn search in NCBI indicated that the significant similarities of 22 sequences were not found, one sequence was involved in tandem repeat, one sequence was involved in DNA transposon, seven sequences had over 90% similarity to *Copia*-like retrotransposon and seven sequences had over 90% similarity to *Gypsy*-like retrotransposon. Additionally, five sequences had over 90% similarity to cDNA and six sequences had over 80% similarity to known wheat genomic DNA sequences that were anonymous. It is interesting that three of the 78 AFLP sequences were coding sequences, however, there were five coding sequences among the 49 MSAP sequences, and their difference was statistically significant (χ^2^<0.05).

## Discussion

### MAALs Induce Genomic and Epigenomic Variations

MAALs of several plant species such as rice, wheat, oilseed rape, sugar beet, cucumber, onion, and soybean have already been created [12], [14]–[19]. These MAALs were mainly used to locate genes or markers on chromosomes, introduce alien genes into plant cultivars, study the cytological behaviours of chromosomes and investigate the effect of single alien chromosome on phenotype. It has been reported that wheat-rye monosomic addition lines can easily induce structural variation of chromosome and high frequency of chromosome translocation [12], [20]. The genomic and epigenetic variations occurred in MAALs and their selfed progeny were seldom reported. In present study, great and different genomic/epigenetic variations were observed among 2R and 5R monosomic addition lines and their selfed progeny without rye chromosome. Furthermore, progeny that only contained 42 wheat chromosomes displayed different genetic/epigenetic variations and it seems that 2RF_3_MY-10 and 5RF_3_MY-1 displayed the highest extent of variations. These results indicated that drastic genomic and epigenetic variations of wheat can be induced by adding single alien chromosome into wheat background. It was interesting that great genetic variations were observed between MY+2RF_2_ and MY+2RF_3_, and between MY+5RF_2_ and MY+5RF_3_, however, drastic epigenetic variations were not detected. The reason why this phenomenon occurred is not clear. It is presumed that there might be no relationship between genetic and epigenetic variations in MAALs. As for the progeny that only contained 42 wheat chromosomes, their drastic genetic/epigenetic variations might be caused by losing the single alien chromosomes. The target products amplified by rye-specific marker from 2RF_3_MY-4 and 5RF_3_MY-1 indicated that the cryptic introgression of rye chromatin into wheat genome could be induced by wheat-rye monosomic addition lines. This may be one of the reasons why MAALs could induce genetic/epigenetic variations. However, 2RF_3_MY-10 displayed outstanding genetic/epigenetic alterations although there were no rye chromatins in this material. This case indicated that introgression of rye chromatins into wheat is not necessary for the drastic genetic/epigenetic alterations. Bento et al. [11] have assessed the genome rearrangements in wheat-rye disomic addition lines and indicated that genomic rearrangement events were not a direct consequence of backcrossing, but resulted from further genome structural rearrangements in the BC plant progeny. In this study, genome reshuffling and epigenetic variation were observed in two continuous generations of wheat-rye monosomic addition lines. The results in present study provided direct evidence to illustrate that MAALs could induce drastic genomic and epigenomic variations. Therefore, monosomic wheat-rye addition lines may be directly used as an effective means to broaden genetic diversity of common wheat.

### Sequences Involved in Genomic and Epigenomic Variations

Restriction fragment length polymorphism (RFLP) analysis has been used to investigate newly synthesized amphiploids and the changes of specific low-copy noncoding and coding DNA sequences have been observed during allopolyploid formation in wheat [21]–[22]. AFLP (*Eco*RΙ-*Mse*Ι combination) and MSAP (*Eco*RΙ-*Hpa*II/*Msp*I combination) methods were used to investigate the allopolyploidy-associated genetic and epigenetic alterations in wheat [23]. Results indicated that changed AFLP sequences corresponded to low-copy DNA and new AFLP bands corresponding to retroelements or DNA transposons were not found, whereas alterations in methylation patterns affected both repetitive DNA and low-copy DNA sequences in almost equal proportions [23]. AFLP and RFLP analyses were used in triticale and results suggested that high degree of coding sequences and repetitive sequences were involved in genomic variation of triticale [1]. These previous results indicated that sequences, which were involved in genetic and epigenetic variations during allopolyploidy, were mainly low-copy noncoding, coding DNA and repetitive DNA sequences. MSAP analysis was used to study the epigenetic variation among wheat-rye 1BL/1RS translocation lines and the alterations of methylation pattern also affected both repetitive and low-copy DNA sequences [24]. In present study, 78 fragments from changed AFLP bands and 49 fragments from changed MSAP bands were cloned and sequenced. For both the AFLP and MSAP sequences, almost half of them showed no significant similarity to known sequences. Among the AFLP sequences whose significant similarities were found, there were few low-copy coding DNA sequences, most of them were retrotransposons and most of the retrotransposons were *Gypsy*-like. Similarly, among the MSAP sequences whose significant similarities were found, there were few low-copy coding DNA sequences, most of them were retrotransposons, however, the numbers of *Copia*-like and *Gypsy*-like retrotransposons were equal proportions. Perhaps, the sequences that were involved in genetic and epigenetic alterations induced by MAALs are different from the ones induced by allopolyploidization. Although few low-copy coding DNA sequences were found in this study, significant difference was observed between ALFP and MSAP coding sequences. In previous studies on allopolyploidization, no great difference was observed between ALFP and MSAP in the variations of low-copy coding DNA sequences [23]–[24]. More evidences are needed to confirm that wheat-rye monosomic addition lines are more likely to induce the epigenetic variation than the genetic variation of coding sequences. In addition, a sequence, which had high similarity to rye genomic DNA sequence, was cloned from a new band in 5RF_3_MY-1. Rye-specific marker amplified products from 5RF_3_MY-1 and GISH analysis indicated that this material only contained 42 wheat chromosomes, therefore, these results confirmed that cryptic rye chromatin has integrated into wheat genome.

In conclusion, single rye chromosome added into wheat background could induce drastic genetic/epigenetic variations and the introgression of cryptic rye chromatins into wheat genome. The introgression of rye chromatins into wheat is not necessary for the drastic genetic/epigenetic alterations. Retrotransposons were mainly involved in genetic and epigenetic variations. Genetic variations basically affected *Gypsy*-like retrotransposons, whereas alterations of methylation pattern affected *Copia*-like and *Gypsy*-like retrotransposons equally. Genetic and epigenetic variations seldom affected low-copy coding DNA sequences. The sequences that were involved in genetic and epigenetic alterations induced by MAALs might be different from the ones induced by allopolyploidization. Monosomic wheat-rye addition lines may be directly used as an effective means to broaden genetic diversity of common wheat.

## Materials and Methods

### Plant Materials

Octoploid triticale MK25 were obtained by crossing between common wheat *T. aestivum* L. ‘Mianyang11’ and rye *S. cereale* L. ‘Kustro’. The parental wheat and rye plants were maintained by strict selfing. Some BC_1_F_2_ and BC_1_F_3_ seeds were obtained by the controlled backcrossing of MK25 with ‘Mianyang11’ followed by self-fertilization.

### Identification of Monosomic Addition Lines

The root mitotic metaphase cells of BC_1_F_2_ and BC_1_F_3_ plants were analyzed through fluorescence *in situ* hybridization (FISH) using repetitive DNA sequence pSc119.2 as probe and genomic *in situ* hybridization (GISH) using genomic DNA of ‘Kustro’ as probe. The repetitive DNA sequences pSc119.2 was labeled with Chroma Tide Alexa Fluor-488-5-dUTP (Invitrogen). The root tips and chromosome spreads were prepared according to the methods described by Han et al. [25]. Probe labeling and hybridization were also operated according to the protocols from Han et al. [25].

### DNA Extraction

Mature seeds of ‘Mianyang11’, ‘Kustro’, wheat-rye 2R, 5R monosomic addition lines and the selfed progeny of the two addition lines were surfaced-sterilized with 70% ethanol and were placed in 15 cm Petri dishes on two layers of soaked filter paper. After germination, plant seedlings were grown in a chamber at 25±2°C under 16 h of artificial daylight and 8 h of darkness for two weeks. Then, leaves were collected from seedlings and they were divided equally. Genomic DNA was extracted from leaves according to the methods described by Zhang et al. [26].

### Investigation of Progeny that Only Contained 42 Wheat Chromosomes Using Rye-specific Marker

To determine whether small rye chromatin has introgressed into the progeny, which only contained 42 wheat chromosomes, a pair of rye-specific DNA primers (5′-GATCG CCTCT TTTGC CAAGA-3′; 5′-TCACT GATCA CAAGA GCTTG-3′) [27] was used to detect the presence of small rye chromatin. The polymerase chain reaction (PCR) was performed according to the procedures described by Katto et al. [27].

### Amplified Fragment Length Polymorphism (AFLP) and Methylation-sensitive Amplification Polymorphism (MSAP) Analyses

*EcoR* I and *Mse* I were used for AFLP analysis, and *Hpa*II/*Msp*I and *EcoR* I were used for MSAP analysis. The AFLP and MSAP procedures were performed according to Zhang et al. [24]. The *EcoR* I, *Mse* I and *Hpa*II/*Msp*I adaptor, the preselective primers, and the selective primer combinations were listed in Table 1.

In both AFLP and MSAP procedures, repeats were carried out and patterns resulting from two independent digestions were compared for each sample. In addition, for both AFLP and MSAP gels, the upper part and the lower part of the gel, where resolution was not satisfactory, were not used for band scoring. Only stable and repeatable patterns were retained for analysis.

### Statistical Analysis

The degree of genomic/epigenetic variations was determined by the analyses of variance, *t*-test and Chi2 test.

### Isolation and Sequencing of AFLP and MSAP Fragments

The polymorphic AFLP and MSAP fragments were isolated from polyacrylamide gels, reamplified by PCR, and sequenced. The procedures were performed according to Zhang et al. [24].

### Sequences Analysis

AFLP and MSAP sequences were analyzed for similarity to known sequences in public databases using BLAST package 2.2 on National Center for Biotechnology Information sever (http://www.ncbi.nlm.nih.gov/Blast).
